# Trafficking in cancer: from gene deregulation to altered organelles and emerging biophysical properties

**DOI:** 10.3389/fcell.2024.1491304

**Published:** 2025-01-20

**Authors:** Julie Patat, Kristine Schauer, Hugo Lachuer

**Affiliations:** ^1^ Cell Biology of Organelle Networks Team, Tumor Cell Dynamics Unit, Inserm U1279 Gustave Roussy Institute, Université Paris-Saclay, Villejuif, France; ^2^ Centre National de la Recherche Scientifique (CNRS), Paris, France; ^3^ Institut Jacques Monod, Université de Paris, Paris, France

**Keywords:** organelle distribution, self-organization, non-random positioning, non-equilibrium steady-state (NESS), lysosomes

## Abstract

Intracellular trafficking supports all cell functions maintaining the exchange of material between membrane-bound organelles and the plasma membrane during endocytosis, cargo sorting, and exocytosis/secretion. Several proteins of the intracellular trafficking machinery are deregulated in diseases, particularly cancer. This complex and deadly disease stays a heavy burden for society, despite years of intense research activity. Here, we give an overview about trafficking proteins and highlight that in addition to their molecular functions, they contribute to the emergence of intracellular organelle landscapes. We review recent evidence of organelle landscape alterations in cancer. We argue that focusing on organelles, which represent the higher-order, cumulative behavior of trafficking regulators, could help to better understand, describe and fight cancer. In particular, we propose adopting a physical framework to describe the organelle landscape, with the goal of identifying the key parameters that are crucial for a stable and non-random organelle organization characteristic of healthy cells. By understanding these parameters, we may gain insights into the mechanisms that lead to a pathological organelle spatial organization, which could help explain the plasticity of cancer cells.

## Introduction

Intracellular trafficking can be described as a dynamic exchange between membrane-bound organelles and/or the plasma membrane of eukaryotic cells. This exchange concerns transmembrane or membrane-bound proteins, such as channels or receptors and their bound ligands as well as macromolecules that are taken up or secreted by the cell. Trafficking is generally classified into two major pathways: the secretory pathway from the endoplasmic reticulum (ER) to the Golgi complex and plasma membrane for neosynthesized macromolecules, and the endocytic pathway from the plasma membrane to endosomes and lysosomes or to the Golgi complex and ER (Scott et al., 2014). Several additional trafficking pathways have been identified, e.g., unconventional secretion from endosomal compartments (Rabouille, 2017) or the various exchanges between different organelles (López-Doménech et al., 2018; Quidwai et al., 2021; Baltrusaitis et al., 2023) Some cell types additionally reveal different types of secretory granules in the cytoplasm that originate from the same pathway as the secretory vesicles however are sorted differently at the Golgi complex.

**FIGURE 1:**
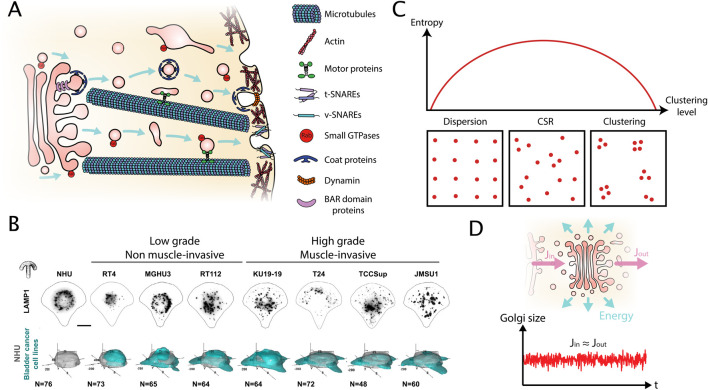
Key properties of intracellular organelle organization resulting from the trafficking activity. **(A)** Schema of ‘intracellular trafficking,’ a dynamic exchange between donor and acceptor membranes of eukaryotic cells. It relies on transport carriers that are characterized by vesicular-tubular intermediates and is supported by a complex machinery made of several families of proteins (Box 1). **(B)** Single cell immunofluorescent images of the ‘lysosome’ organelle in normal human urothelium (NHU) cells as well as cell lines representing ‘low grade,’ non-muscle invasive and ‘high grade,’ muscle invasive bladder cancer on crossbow-shaped micropatterns to facilitate their comparison (upper panel). Quantification of lysosome positioning by 3D density maps, a method from the spatial statistics toolbox, of N > 45 cells from (lower panel) Mathur et al. (2023). **(C)** Relationship between the entropy associated to a point pattern and the clustering level of the point pattern. The inserts illustrate the three archetypical point patterns: dispersion, Complete Spatial Randomness (CSR) and clustering. Because there are many ways to obtain a CSR pattern, but less possibilities for a dispersed or clustered one, the entropy is maximal in case of CSR. **(D)** The Golgi complex receives an inward flux (J_in_) and an outward flux (J_out_). Then the inward and outward fluxes are equal, the derivative of the Golgi mass is close to zero, and the Golgi size does not change significantly. Thus, considering the Golgi as stationary is a good approximation at short time scales. However, because the active fluxes dissipate energy, the system is out of the thermodynamic equilibrium, it defines a Non-Equilibrium Steady State (NESS).

Trafficking relies on transport carriers that are characterized by vesicular-tubular intermediates and is supported by a complex machinery made of several families of proteins (Figure 1A; Box 1). The budding of vesicular-tubular transport carriers from a donor membrane requires the recruitment of *membrane-deforming proteins*, *membrane-constricting proteins* and *coating proteins*. Once the scission with the donor-membrane is completed, vesicular-tubular transport carriers are transported along the *cytoskeleton* with the help of *motor* proteins and fuse with a receiving membrane. Different types of *adapter molecules* connect motor proteins to transport carriers during trafficking. The fusion with a receiving membrane is initiated by *tethering molecules* and facilitated by a *fusion machinery*. The different steps of trafficking are regulated by *small GTPases* of different families that by themselves are regulated by Guanine Nucleotide Exchange Factors (*GEFs)* and GTPase Activating Proteins (*GAPs)*.

Many genes coding for the trafficking machinery are deregulated in cancer. Yet, despite decades of study, there is no general description of their contribution to carcinogenesis: indeed whereas some members of the trafficking machinery can be downregulated in some cancers, they are upregulated in others, e.g., Caveolin-1 expression is upregulated in pancreatic and prostate cancer while downregulated in lung and breast cancer (Chatterjee et al., 2015; Thompson et al., 2009; Volonte et al., 2017; Wang et al., 2020). The last decades have shown that trafficking can adopt to different perturbations and change in response to cues from the environment and due to genetic or epigenetic modifications. The adaptability of trafficking pathways supports cell plasticity, a major characteristic of cancer cells (Muthuswamy and Bin, 2012). Very little trafficking proteins have been therapeutically targeted due to their critical physiological roles (Di Marco et al., 2020). To gain a deeper understanding of the role of trafficking in cancer, we propose to zoom out to a higher level: Trafficking pathways shape intracellular patterns, which we recognize as organelles. Indeed, the ability to create patterns spontaneously is one outstanding conserved feature of life. Jacques Monod even included this criterion in his definition of life under the name of autonomous morphogenesis (Monod, 1970). Pattern formation in living matter appears at many scales and has been well described on the organism level, e.g., during development (Belintsev et al., 1987). Intriguingly, much less is known about intracellular pattern formation of organelles through trafficking processes. Here, we will discuss organelle patterns as a superior scale of trafficking in the context of cancer.

## Intracellular organelle patterns are different between healthy and transformed cells and correlate with cancer aggressiveness

Although widescale efforts have been made to establish atlases of genomic and proteomic alteration in cancer, no atlas of organelle-level changes has been profiled till date. Systematic studies on intracellular organelles are difficult, because, on the one hand, *in vivo* approaches are limited by the access of the samples and the lack of subcellular resolution, and on the other hand, *in vitro* cultured cells display a dynamic shape and strong morphological cell-to-cell variation. To address these limitations we and others have employed novel bioengineering and image analysis approaches: normalized cell culture conditions on adhesive micropatterns have been combined with spatial statistics approaches based on probabilistic mapping (Schauer et al., 2010; Jerabkova-Roda et al., 2023), or novel artificial intelligence (AI) methodology (Wang et al., 2023) has been implemented in order to quantify organelle spatial organization and topology. Using these technologies, the hypothesis was tested that organelle patterns in cancer cells are different from those of normal cells and change during aggressiveness (Mathur et al., 2023; Jerabkova-Roda et al., 2023; Wang et al., 2023). The rationale of this was that the observed vast alterations in trafficking processes in cancer cells will give rise to altered patterns of organelles as organelle emerge from trafficking.

First, using a bladder cancer model and well-controlled culture conditions, lysosomes of normal human urothelium (NHU) were compared to invasive and non-invasive bladder cancer cell lines that represented low-grade and high-grade bladder cancer, respectively. Lysosomes are dynamic, acidic organelles for cell clearance and recycling of macromolecules and act as cellular hub for metabolism and signaling. While in NHU cells lysosomes were positioned centrally, they were peripherally dispersed in bladder cancer cells with a stronger phenotype in ‘high-grade’ cell lines (Mathur et al., 2023) (Figure 1B). Similarly, lysosome positioning was altered in patient-derived melanoma cells and patient biopsies, scaling with and supporting melanoma aggressiveness (Jerabkova-Roda et al., 2023). Interestingly, the team of M. Barroso recently integrated artificial intelligence (AI) and imaging quantification to analyze organelle spatial distribution in the breast cancer model. They found that organelle topology allows for a highly precise differentiation between cell lines of different subtypes and aggressiveness (Wang et al., 2023).

Together, these results indicate that alterations of organelle patterns correlate with transformation and aggressiveness of cancer cells. Moreover, when lysosomal perinuclear clustering was induced experimentally in patient-derived melanoma cells, we observed significant reduction of invasive outgrowth in *mouse* and *zebrafish* models (Jerabkova-Roda et al., 2023). This study provided a direct demonstration that lysosomal positioning controls cell invasion, illustrating the importance of organelle adaptation in carcinogenesis. Thus, we argue that the patterning of organelles, which represents trafficking collectively, could potentially not only be used as a biomarker in the future, but should also to be considered to better understand, describe and fight cancer. In the next sections, we focus on a physics description of the organelle landscape to identify critical parameters for a stable and non-random organelle organization characteristic of healthy cells.

## How to describe organelle organization?

Based on experimental data, we argue that organelle organization has three key properties: non-random distribution, Non-Equilibrium Steady-State (NESS) and self-organization.

### Non-random distribution

When organelles are observed in cells, a fundamental question is whether their positioning is random or not. A distribution of organelles can be simplified as a point pattern if the typical size of the object is small compared to the distances between objects (Schauer et al., 2010). In spatial statistics, a “uniformly random” distribution of points is described as a Complete Spatial Randomness (CSR). Formally, a CSR is defined by the fact that i) each point’s location is independent of the other points, and ii) the probability of finding a point in a sub-region only depends on the ratio between this sub-region volume (or area in 2D) and the total volume (or area). Diggle (2013) defined two primary deviations from this CSR pattern: i) clustering (i.e., aggregation) and ii) dispersion (i.e., points repel each other mutually forming a regular grid), (Figure 1C). Spatial statistics provides tools to quantify the level of organization of point patterns (Box 2) (Dixon, 2014). Interestingly, these tools have been used extensively for Single Molecule Localization Microscopy (SMLM) (Khater et al., 2020) or to describe exocytosis distributions (Sebastian et al., 2006; Yuan et al., 2015; Urbina et al., 2018; Lachuer et al., 2023), but were rarely applied to organelles with few notable exceptions (Schauer et al., 2010; Ba et al., 2018).

A CSR pattern is expected as a result of the diffusion that maximizes the entropy of the system (Figure 1C). However, many examples of a non-random organization are found in cells: for instance, organelles such as lysosomes and mitochondria often accumulate perinuclearly (Collins et al., 2002; Jongsma et al., 2016). A systematic analysis revealed that diverse organelles (early endosomes, lysosomes, multivesicular bodies, etc.) have a unique and well-defined distribution (Schauer et al., 2010; Duong et al., 2012). More recently, using spatial statistics, it has been demonstrated that lysosomes are not randomly distributed at the whole cell scale (Ba et al., 2018). It demonstrates that mechanisms are at place that actively counterbalance diffusion to shape the organelle landscape. The resulting non-random organization of cellular structures can be understood as an adaptation to support a function: a spatial-organization function relationship (Bergeijk et al., 2016; Vaidžiulytė et al., 2019). Indeed, it has been widely documented that organelles dynamically adopt their intracellular positioning to various stimuli, such as pH, nutrient availability or their microenvironment. For example, the spatial organization of lysosomes is influenced by cholesterol concentrations (Rocha et al., 2009), intracellular pH (Walton et al., 2018) or extracellular matrix elasticity (Wang and Galli, 2018), (see Table 1 for a non-exhaustive list). The underlying cellular mechanisms and proteins that regulate organelle positioning have been reviewed extensively (e.g., endolysosomal compartment (Bonifacino and Neefjes, 2017); Golgi complex (Brownhill et al., 2009); mitochondria (Kruppa and Folma, 2021). Non-random organization allows for high local concentrations and the possibility of segregating incompatible biochemical reactions.

**TABLE 1:** Conditions under which changes in organelle positioning have been observed. Here we provide a non-exhaustive list of conditions during which organelle positioning changes have been documented.

Conditions under which organelle change positioning	Organelle	References
Cell cycle	All organelles	Gkini et al. (2024); Bergeland et al. (2001); Tang et al. (2010)
pH changes	Lysosomes	Parton et al. (1991); Heuser (1989); Johnson et al. (2016)
Calcium concentration changes	Lysosomes, mitochondria	Li et al. (2016); Macaskill et al. (2009); Quintana et al. (2006)
ER stress/unfolded Protein Response is activated (UPR)	Lysosomes Mitochondria ER	Bae et al. (2019)
Nutrient abundancy changes	Lysosomes	Korolchuk et al. (2011)
Hypoxia condition/oxidative stress	Lysosomes, ER, Golgi-complex	Walton et al. (2018); Nakahara et al. (2023); Sasazawa et al. (2022)
Presence of growth factors	Lysosomes Mitochondria	Jia and Bonifacino (2019); Minin et al. (2006)
Cholesterol concentration changes	Lysosomes	Rocha et al. (2009); Johansson et al. (2007)
Migration	Mitochondria Golgi complex Nucleus	Mosier et al. (2023); Kupfer et al. (1982); Gomes et al. (2005)
Substrate stiffness	Lysosomes, Mitochondria	Wang et al. (2018); Chen et al. (2021); Daga et al. (2024)

Intriguingly, the non-random positioning of organelles could stem from the non-independent nature of organelles that need connectivity. It has emerged during the last few years that the organelle landscape is defined by numerous organelle membrane contact sites (MCS). This leads us to think of organelles as a network that constantly communicates, exchanges material, and changes topology. Indeed, it has been demonstrated that this organelle network is not randomly established (Valm et al., 2017), confirming a non-random spatial organization of each organelle. For example, it has been reported that mitochondria and ER structures are close to secretory sites, probably because they play a role in regulating exocytosis by Ca2+ signaling (Villanueva et al., 2014; Griesche et al., 2019). Interestingly, a feedback regulation between MCS and cellular organization has been proposed. For example, the spatial organization of lysosomes determines its MCSs with ER (Ba et al., 2018), and in return, MCSs are able to control the positioning of lysosomes (Pu et al., 2016; Jongsma et al., 2016; Bonifacino and Neefjes, 2017; Neefjes et al., 2017; Cabukusta and Neefjes, 2018).

### Non-equilibrium steady state

Organelle patterns are often described as a steady state, meaning that they remain relatively constant over time (Figure 1D), (Pelham, 1996; Schauer et al., 2010). However, this steady state statement is limited, because the cell organization shows small random fluctuations around a steady state. In addition, at longer time scales, the cell organization cannot be seen any more as a steady state. Indeed, cell organization dynamically changes, for instance, during the cell cycle or in response to a variation in the environment (see Table 1). Therefore, the steady state statement is a good approximation at short time scales, i.e., time scales shorter than the cell cycle (typically <10 h in eukaryotic cells). For example, it has been demonstrated that lysosomal organization in classical petri-dish conditions is at a steady state during a time window of several minutes to hours (Pelham, 1996; Ba et al., 2018; Guardia et al., 2019; Duong et al., 2012). At steady state, spatial parameters such as distance to the nucleus, inter-organelle distance, and nearest-neighbor distance (and Ripley’s K function, see Box 2) have a constant distribution. Additionally, the organelle connections, especially MCS defining the organelle network, have been found to be stable for at least several minutes (Valm et al., 2017). Importantly, steady state should not be understood as the immobility of individual organelles but as the conservation of global organization despite individual organelle movements consuming energy. Due to these dynamics based on trafficking processes, the system is out of equilibrium: it consumes energy. Such a system is called a Non-Equilibrium Steady State (NESS) that is typical for biological samples.

Interestingly, after a reversible perturbation of the endomembrane organization, the cell spontaneously converges to its physiological steady state organization. For example, after a reversible coupling of lysosomes to classical anterograde kinesin or to the unconventional retrograde kinesin 14 (KIFC1), forcing respectively a peripheral or central clustering, lysosomes can spontaneously re-find their original steady state organization in a dozen of minutes (Guardia et al., 2019). Similar effects have been observed with changes of environmental cues, e.g., such as pH. After a perturbation, the system converges toward a reference state. In accordance to experimental observations, computational models of organelle organization often converge to a steady state (Dinh et al., 2006; Birbaumer and Schweitzer, 2011; Gou et al., 2014). In other models, the steady state is expected and used as a hypothesis that significantly facilitates the resolution of differential equations (Higuchi et al., 2014; Lin et al., 2016).

### Self-organization

Self-organization is the emergence of a spatio-temporal organization resulting only from the interactions of the individual components (Karsenti, 2008). Contrarily to self-assembly, self-organization involves energy consumption (Misteli, 2001; Wedlich-Söldner and Betz, 2018). The importance of self-organization has been discussed in the context of cellular architecture (Misteli, 2001; Wedlich-Söldner and Betz, 2018). Authors argue that forming distinct organelles is self-organized. For example, the Golgi complex spontaneously reassembles after mitosis (Wei and Seemann, 2017) suggesting a self-organization property also predicted by theoretical models (Vagne et al., 2020; Tachikawa, 2023). It is also thought that MTOC are self-organized (Pereira et al., 2021) although a full centriole reconstruction has not been achieved yet *in vitro*, successful attempts have been reported for critical components of centrioles (Guichard et al., 2017). Interestingly, once assembled, the MTOC can autonomously find the center of the cytoplasm (Malikov et al., 2005) demonstrating the self-organization of its spatial localization. Future work in artificial cells could formally test the hierarchy in self-organization processes between the organelle network and the cytoskeleton and identify the minimal components that are necessary to recapitulate the organelle spatial organization in an *in vitro* system.

## How is a non-random steady state self-organization within cells achieved and maintained?

One main challenge is understanding how cell compartments’ non-random steady state self-organization is achieved and maintained from intracellular trafficking. In this respect, no single gene, or not even one gene regulatory network, organizes the cell, instead, organelle organization emerges from many genes. Thus, a comprehensive understanding of intracellular organization requires the investigation of the interactions between genes and the resulting emerging laws. In the next section, we propose to consider biophysical models that describe pattern formation. We review how these models were applied in the context of trafficking proteins to explain pattern formation of organelles. Due to their bacterial origin, mitochondria are special organelles that are distinct in their participation in the cellular trafficking pathways (Baum and Baum, 2014) and can undergo fusion/fission. Similarly, membrane-less organelles such as stress granules, P-bodies, etc., which are biological condensates formed by liquid phase separations, rely less on classical trafficking machinery described in Box 1. Thus, their pattern formation could be described by different models than whose used for instance for endosomes or Golgi complex.

### Chain of interactions

**FIGURE 2:**
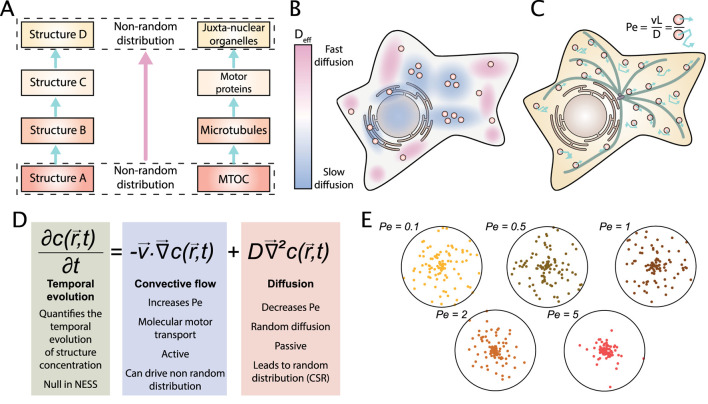
How is a non-random steady state self-organization within cells achieved and maintained? **(A)** Chain of interactions: Structures with a non-random distribution can transmit their spatial organization via molecular interactions. For example, the centrosomal Microtubule Organizing Center (MTOC) typically nucleates microtubules in an aster geometry. Motor proteins (kinesins and dyneins) bind organelles and attach them to the microtubule network, transmitting its spatial organization. Because of the aster geometry, microtubule concentration is higher close to the MTOC entailing a juxta-nuclear organization of organelles. **(B)** Heterogeneous diffusion: because the cytoplasm is heterogeneous and anisotropic (in terms of viscosity as well as molecular activity), there are regions of high and low diffusivity, characterized by different effective diffusion coefficients D_eff_ of diffusing particles. Regions of different diffusivity can create local clustering patterns. **(C)** Convective flow: Energy is used to create a convective flow. For example, motor proteins use ATP to transport particles on microtubules. A system dominated by convective flow is characterized by a high Péclet number and is susceptible to create spatial patterns. Thus, energy consumption balances random distribution driven by diffusion. **(D)** The Fokker-Planck equation represents the temporal evolution of the concentration c of a particle as a function of the convective flow controlled by the speed v and the Brownian motion controlled by diffusion coefficient D. In NESS, the temporal evolution is null by definition. **(E)** We model the particle concentration c from steady-state solution of the Fokker-Planck equation with a centripetal flow. Five results are represented obtained in simulations for 2D disk cells and different Péclet Numbers. The degree of clustering correlates with the Péclet Number.

The interactions between molecules can lead to distinct forms of clustering: a cellular component that has a stable distribution will dictate its spatial structure to its bonded partners. This process creates a chain of interactions where spatial structure is conserved (Figure 2A). For example, Pangarkar et al. have studied the distribution of early endosomes in mammalian cells. They report a juxta-nuclear accumulation of endosome despite an unbiased bidirectional movement (Pangarkar et al., 2005). Authors explain this result by the aster structure of microtubules. Because the local density of microtubules is higher at the center of the aster, the local density of binding sites for endosome is also higher. This creates a central accumulation. This could also explain why a perinuclear cloud of lysosomes is observed (Jongsma et al., 2016). Thus, microtubules function as a brick for a chain of interactions that transfers spatial structure. Similar behavior has been proposed for the ER to organize spatial distribution of P-bodies or lipid droplets (Lee et al., 2020; Guyard et al., 2022). This mode to create non CSR pattern can be seen as a special case of a chain of interactions where a network transmits a spatial pattern to all its interacting proteins and organelles.

Plasma membrane subdomains seem also to function as a common first brick for chains of interactions (Dykstra et al., 2003; Simons and Toomre, 2000). For example, it has been shown that H-Ras clusters in lipid rafts, similar to the GLU/GLUT4-cluster at the plasma membrane that is lipid-raft dependent, probably because of a direct interaction (Prior et al., 2003; Gao et al., 2017; Yan et al., 2018). Interestingly, spatial statistics of proteins associated with lipid rafts have been used to test the validity of different raft models (Plowman et al., 2005). Chains of interactions could be a major mechanism of spatial organization in the cell. However, this mechanism is not sufficient to explain non-CSR distribution, because it postulates that the first element of the chain has already a special distribution. Moreover, some patterns seem totally independent of other proteins. For example, the plasma membrane-anchored kinase Lck clustering seems to be independent of the association with molecular networks or with lipid domains (Rossy et al., 2013). Moreover, the cell can rely on the geometry of its micro-environment as a first instructing brick to create a chain of interactions. For example, it has been observed that a grid of rigid micro-pillars can spatially organize the formation of podosome-like structures in dendritic cells (Rathar et al., 2024).

### Heterogeneous diffusion

All small particles such as molecules, protein complexes, and small organelles are subject to thermal diffusion (also called Brownian motion). The diffusion coefficient D characterizes the diffusion process and is given by the Stokes-Einstein equation $D=\frac{k_{B}T}{6\pi \eta R}$ with k_B_ the Boltzmann’s constant, T the temperature, η the dynamic viscosity and R the radius of the particle (assumed to have a spherical section). For example, a lysosome that is only subjected to diffusion will travel microns in few seconds. Because there exists no preferential direction, thermal diffusion leads to a Complete Spatial Randomness (CSR) distribution. Note that the thermal diffusion is a consequence of the second law of thermodynamics: the entropy of an isolated system can only increase and thus drives to CSR. However, the cytosol is an active environment with molecular crowding (Hall and Minton, 2003) that significantly affects diffusion (Luby-Phelps et al., 1987). Thus, the Stokes-Einstein equation could under-estimate the observed diffusion due to active fluctuations stirring the cytosol (Brangwynne et al., 2009). Moreover, cytosol is not an isotropic and homogeneous environment as postulated in simple diffusion models. Especially, a non-uniform viscosity in the cell has been reported (Kuimova, 2012; Mandal et al., 2016; Lonhus et al., 2021). In areas with a higher viscosity, the diffusion should be slower according to the aforementioned Stokes-Einstein equation (Garner et al., 2023). This could lead to local clustering only due to heterogeneous diffusion (Raja et al., 2024). Under this condition of non-uniform viscosity in space, diffusion does not create a homogeneous pattern but clustering in high viscosity area (Figure 2B). Heterogeneous diffusion is particularly important at the plasma membrane (Johannes et al., 2018). For example, it has been reported that LAT protein (Linker for Activation of T cells) diffuses slower in ordered-lipid domains (Owen et al., 2012), and thus accumulates in these domains. Even if diffusion heterogeneity is important, little is known about the consequences, probably because of the difficulty to perform experiments.

### Convective flow

The passive mobility from thermal diffusion of small particles can be counteracted by an active convective flow. In cells, the convective flow mobility is achieved by *molecular motors* (kinesin, dynein and myosin) and creates a directed flow. The speed v of the flow characterizes the convective transport. Whereas diffusion leads to a Complete Spatial Randomness (CSR) distribution, convective flow can create specific patterns. Thus, convective flow could allow the cell to achieve a non CSR distribution. The archetypal example of this is the anterograde secretion pathway (Figure 1D). The relative importance of convection flow on diffusion is quantified by the Péclet number Pe (Pe = vL/D where L is a characteristic length) (Figure 2C). Thereby, a low Péclet number corresponds to a cell with a low rate of directional transport leading to a CSR distribution, and a high Péclet number leads to the pattern formation. The resulting distribution of particles can be described by the Fokker-Planck equation (also called advection-diffusion equation) (Figure 2D).

## Combinatory models: reaction-convection-diffusion model

The different ways to create patterns are not mutually exclusive, but rather are working in combination with a strong interplay between them. Indeed, particles such as protein complexes or organelles could shuttle between heterogeneous diffusion in the cytosol, tethering to the *cytoskeleton* due to a chain of interactions, and convective motion driven by *molecular motors* walking on *cytoskeleton*. Building on the idea that particles can switch from one state to another, models have been proposed that couple different states leading to the addition of reaction terms. These seminal models for spatial distribution of particles are reaction-diffusion-convection models. Often they can be reduced to a simpler diffusion-convection model thanks to an approximation on the different time scales of these processes. The simplification relies on the hypothesis that the reaction timescales are small compared to convection/diffusion timescales. For example, an organelle distribution model with three states: i) diffusion ii) microtubule (+) transport and iii) microtubule (−) transport that can be modeled by a set of Partial Differential Equations (PDEs), (Figure 2D). The first part is driven by thermal fluctuations and characterized by its diffusion coefficient. In contrast, the second and third parts are mainly achieved by energy-consuming processes relying on molecular motors that create an emerging convective flow characterized by a velocity. As an illustration, simulated organelle distributions are shown based on a convection-diffusion equation in 2D disk cells for different Péclet numbers (Figure 2E). This simulation illustrates that the clustering increases with the Péclet number. Some other scholars rely on reaction-diffusion models, since the seminal Turing paper of 1952 (Turing, 1952). The Turing model can reproduce complex self-organized morphogenesis patterns (Kondo and Miura, 2010) in a wide variety of situations (Kondo and Asai, 1995; Jacobo and Hudspeth, 2014; Blagodatski et al., 2015; Sekimura et al., 2015; Fofonjka and Milinkovitch, 2021). It has been recently proposed that the Turing model is a general principle of cellular self-organization at the molecular scale (Halatek et al., 2018). However, the Turing model has a robustness problem, because diverse variations in parameters, such as initial conditions, presence of noise, or delay can vanish the pattern (Bard and Lauder, 1974; Murray, 1982; Maini et al., 2012) while experimental work indicates that organelle landscape is robust (Duong et al., 2012; Guardia et al., 2019). Moreover, neglecting the convective flow seems accurate for chemical systems, but is too simplistic for organelle patterning that clearly relies on motor-driven transport.

A lot of work on the experimental and theoretical part of the analysis of organelle distribution has been performed in hyphae of the filamentous fungi *Ustilago maydis* (Lin and Steinberg, 2017; Gou et al., 2014; Higuchi et al., 2014; Lin et al., 2016). The efficiency of modeling in hyphae is due to its constant and simple cylindrical morphology. Some modeling has also been performed on animal cells. For example, the distribution of endosomes (Pangarkar et al., 2005) and lipid droplets in embryonic *Drosophila* cells (Maly, 2002), as well as endocytosed virus (Lagache and Holcman, 2008; Lagache et al., 2009) was efficiently modeled by the Fokker Planck equation. Thus, convection-diffusion models are able to reproduce the spatial organization of organelles in a large diversity of situations, and a general model of organelle distribution has been proposed (Dinh et al., 2006). According to the chosen parameters, this model generates different kind of organizations. Surprisingly, this simple model can produce patterns similar to a good range of experimental data.

Some papers use the full reaction-diffusion-convection model (Smith and Simmons, 2001; Dinh et al., 2005). For example, 4-states models using reaction-diffusion-convection propose that melanosome distribution is controlled mainly by microtubule binding rate (Slepchenko et al., 2007). Another paper proposes that endosome clustering is controlled by a Péclet number and an organelle interaction coefficient (Pangarkar et al., 2012). The common point of these models is that they do not generate instability or oscillations, confirming the stabilization effect of convection.

## Can physical models potentially reveal critical parameters of carcinogenesis?

**FIGURE 3:**
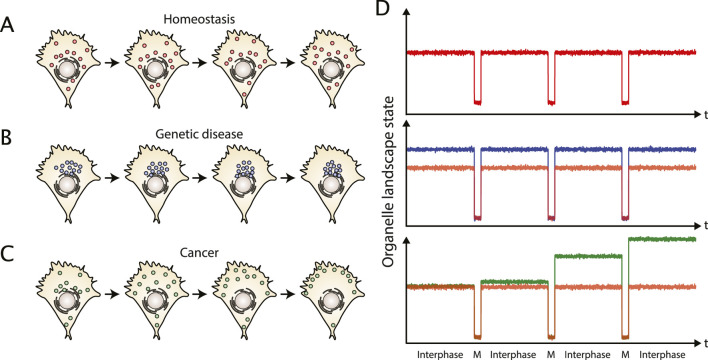
Organelle landscape dynamics at long time scales in healthy and pathological contexts. **(A)** The statistical properties of the organelle landscape in a healthy cell are preserved over several divisions. **(B)** Genetic diseases based on finite mutations can exhibit a pathological organelle landscape, but it is still constant over divisions. For example, Huntington’s disease is associated with a perinuclear clustering of lysosomes. **(C)** In cancer, the spatial organization is not preserved over divisions. While the cancer cell accumulates mutations, the organelle landscape is perturbed accordingly. **(D)** The organelle landscape of a healthy cell (red) is in a steady state during interphase with random fluctuations, while at longer time scales, the system can be seen as oscillatory, switching from an interphase state to a mitotic (M) state. Cells with a genetic disease (blue) are at a different steady state than healthy cells (depicted by the red curve for comparison), but reveal similar oscillations at longer time scales. Cancer cells (green) typically accumulate mutations that keep changing their organelle landscapes over divisions. The system cannot be regarded as oscillating anymore contrarily to healthy cell (depicted in red for comparison).

Based on recent observations of organelle changes in cancer we argue that carcinogenesis could be seen as a loss of a reference steady state organization. In this context, the increasing mutations in diverse genes, including trafficking proteins, either could support the loss of the reference state or could compensate this loss. Interestingly, organelle steady state dynamically changes during cell division (see Table 1): it seems to oscillate along the cell cycle when observed at longer time scales (Carlton et al., 2020). Notably, daughter cells re-find a comparable steady state as the mother cell after cell division under homeostasis (Figures 3A-D). Contrary, cancer cells seem to change at these time scales (Figure 3C). Thus, whereas healthy, differentiated cells could be described as being in an oscillatory state at long time scales, cancer cells can neither be considered as steady nor oscillatory (Figure 3D). Cancer cells seem instead to diverge versus a dynamic state that we define as plasticity.

Notably, a chaotic regime seems to be easily achieved in biological systems, as both oscillations and chaos can emerge in a non-linear system with time delay (Mackey and Glass, 1977). Oscillations/chaos have been observed in many biochemical reactions (Sel'kov, 1968; Aon et al., 2011; Benincà et al., 2015; Olsen and Degn, 1977; Decroly and Goldbeter, 1982; Olsen and Degn, 1985; Baier and Sahle, 1998). However, chaotic regimes have not yet been observed for the organelle landscape. Future investigations could reveal whether cells have mechanisms to avoid chaotic regimes altogether, or if these regimes exist but have not been observed due to the lack of quantitative measures at the appropriate time scales. This question is especially relevant for cancer cells that randomly accumulate mutations, making their organelle landscape potentially unstable. Alternatively, intra-tumoral natural selection could stabilize whose organelle landscapes that correlate with the most aggressive cell behavior.

Modeling results illustrate that cell organization can be described by general physical principles. Thanks to this biophysical description, loss of organization could be considered as a stability problem of physical models. Interestingly, modeling allows to identify properties that contribute to stability. For instance, it is well known that the Turing model has a robustness problem (Bard and Lauder, 1974; Murray, 1982; Maini et al., 2012), but can be stabilized by the addition of convection. As convection often is represented by molecular motor activity, we speculate that the increased expression of motor proteins seen in melanoma could be interpreted as a compensation for the loss of stable organelle positioning at long time scales that is observed in aggressive melanoma cells (Jerabkova-Roda et al., 2023). Alternatively, active supply with ATP due to increased energy production, as often observed in cancer cells, could increase the convective flow indirectly.

Further, from the theoretical part, boundary conditions can also have an impact on the stability of the system. For example, it has been reported that Dirichlet boundary condition (i.e., fixed concentrations at boundaries) enhances stability of the Turing model (Dillon et al., 1994). It will be interesting to explore whether cancer cell interactions with their microenvironment including other cell types could represent some boundary conditions for organelle distribution. Indeed, the effects of boundary conditions have not yet been extensively studied in the context of organelle distribution.

Finally, it could be considered that alterations of specific gene families contribute in different ways to instability. For instance, Ras alterations could potentially contribute to organelle distribution changes through the induction of a strong chain of interactions, whereas motor proteins could contribute through changes in convection. Changes in families that regulate lipid composition of membranes or cell crowding could alter the diffusion behavior of membrane proteins or cellular macromolecules, and could contribute to organelle alteration through the diffusion term.

Different from cancer, genetic diseases based on finite mutations, such as lysosomal storage disorders or Huntington’s disease (Erie et al., 2015), could be characterized by a transition from one steady state organization versus a new pathological organization sustaining harmful functions (Figures 3B, D). In this case, the loss of the physiological steady state organization could be described as a modification in the parameters of these models. For example, mutations in motor proteins would change the Péclet number or their kinetic rate changing the reaction terms in the models without leading to instability.

## Conclusion/outlook

Here, we zoomed out from the molecular functions of trafficking proteins and looked at one level above: the intracellular patterns of organelles that represent the integrated behavior of cellular trafficking activity. The rationale of this is that cancer cells reveal an altered intracellular organization of organelles at long time scales, and we argue that these changes in organelle patterning need to be considered to better understand, describe and fight cancer. Based on the fact that organization can be described by biophysical models, we propose to consider physical emerging properties such as instability of organization as an interesting but unexplored property of cancer cells. In the future, it could be propitious to perform rigorous stability analyses of different models to further identify sources of loss of the stable organization as seen in cancer cells at long time scales.

Of course, there are several limits of the discussed Partial Differentiation Equitation models due to several assumption that are made for simplification. These include the i) well-mixed system approximation that cytosol is homogeneous, ii) linear kinetics hypothesis that binding/unbinding processes can be modeled by first order reactions and iii) the choice of boundary conditions that are often under-estimated. Alternatively, some authors proposed an agent-based approach to model organelle dynamics (Mayorga et al., 2018). In agent-based modeling, the system is modeled by a set of discrete entities called agents. Each agent behaves according to a set of predefined rules. Emerging effects can appear from individual behavior of agents. These models are stochastic and more apt to describe local regulations (Dalmasso et al., 2017; Dinh et al., 2007) or feedbacks (Mayorga et al., 2017). Unfortunately, these models have not been used extensively to investigate the spatial distribution of organelles. Future work should show if they are consistent with models based on PDE, how they compare to these and which properties of complex systems we can learn from them. These models should be investigated and compared in normal versus pathological cell conditions such as cancer.

Investigating the dynamic intracellular landscape of organelles, showing fascinating pattern alterations, could allow the establishment of novel biomarker. Potentially, alterations in organelle patterns could show less heterogeneity than gene/protein expression profiles of different cancers. This could potentially facilitate the prediction of cancer progression or cell responses to drug treatment and resistance.

Finally, organelle patterns could also help reveal the cancer microenvironment and identify cancer cell addictions that make them more vulnerable to treatment than healthy cells. Intriguingly, organelle positioning strongly correlates with environmental cues (see Table 1). Therefore, changes in organelle landscapes observed in cancer could indicate which alterations are found in their microenvironment. For instance, peripheral lysosome trafficking in tumor cells was found to result from acidic extracellular pH, inducing cathepsin B secretion and tumor invasion. This behavior was reversed by inhibitors of sodium-proton exchangers (NHE) that induced a time-dependent retrograde aggregation of lysosomes (Steffan and Cardelli, 2010). More recently, in Glioblastoma stem-like cells (GSCs), organelle alterations have been linked to cholesterol addiction that revealed a vulnerability of glioblastoma to cholesterol-lowering drugs such as statins, particularly in conditions in which organelle alterations were aggravated (Maghe et al., 2024). Thus, interfering with cancer cell addictions, revealed by changes in cellular organization, could emerge as an effective strategy for cancer cell elimination.

The contribution of organelle dynamics in cell homeostasis and disease is still understudied due to lack of resolution, accessibility and cell-to-cell heterogeneity observed in classical cell models. Therefore, novel tools from mathematics, physics and engineering are urgently needed to study organelles under controlled culture conditions in higher dimensions. The focus on intracellular organelles constitutes a novel effort addressing cell perturbation beyond individual gene modification that will open exciting perspectives in the understanding, diagnostics and therapeutics of multifactorial diseases such as cancer.

Box 1 **| Proteins of the trafficking machinery**

Coat proteins: The main studied coat protein is clathrin (Ungewickell and Branton, 1981). Three heavy and three light chains of clathrin form a triskele (Heuser and Steer, 1989), whose repeat assembly leads to membrane curvature. The resulting hexagonal cage around the invaginated budding structure is called clathrin coated pit. Caveolins are around 20 kDa proteins known to be involve in the formation of cavolae, which are membrane invaginations enriched in proteins and distinct lipids (e.g., cholesterol, sphingolipids). Two caveolin isoforms are ubiquitously expressed, Cav1 and Cav2, whereas Cav3 is only found in muscle cells. Caveolins interact with cavins to bend membranes and to form the invaginating caveolae (Vinten et al., 2005; Hill et al., 2008). To transport cargos from the Golgi complex to the ER, the coat protein complex I (COPI) is recruited to Golgi complex membranes. This complex is composed of seven subunits: α-COP, β-COP, β′-COP, γ-COP, δ-COP, ε-COP, and ζ-COP. The induced extreme curvature of the membrane will allow the COPI coated vesicles to detach from the Golgi membrane (Tomás et al., 2010). The coat protein complex II (COPII) generates vesicles mediating protein transport from the ER to the Golgi complex. COPII complex is composed of SEC23, SEC24, SEC13, SEC31 and the small GTPase SAR1A. COPII coat is responsible for direct capture of cargo proteins and for the physical deformation of the ER membrane (Farmaki et al., 1999).

Cytoskeleton: Actin is the most abundant protein in the cell and forms filaments (F-actin for filamentous) by the polymerization of globular actin (G-actin for globular). Actin filaments are oriented with barbed end (called plus end) bound to ATP making them more stable, therefore having a faster rate of polymerization. On the other hand, the pointed end (minus end) bound with ADP making them more susceptible to disassemble (Kirschner, 1980). Microtubules form the other dynamic cytoskeleton network. These filaments made of dimers of ***α*** and ***β*** tubulin, radiate from the Microtubules Organizing Center (MTOC). The major MTOC is the centrosome, a structure composed of two centriole surrounded by pericentriolar material, but non-centriolar MTOC exists such as the ones at the Golgi apparatus (Zhu and Irina, 2013; McGill and Brinkley, 1975). Inside the centrosomal MTOC, **γ** -tubulin and other proteins form a ring to start the nucleation of ***α***-***β*** tubulin dimers (Aher et al., 2024). The minus end of microtubules reveals the ***α***-tubulin, whereas ***β***-tubulin is prominent at the positive, dynamic end.

Motor proteins: There are different families of motor proteins that bind to either the microtubule cytoskeleton, called kinesins and dyneins, or the actin cytoskeleton, called myosins. Kinesins mostly facilitate movement towards the plus end of microtubules (Hirokawa et al., 1991) with the exception of the retrograde kinesins 14 (KIFC1,2,3), whereas dyneins facilitate movement towards the minus end of microtubules, thus often the centrosomal MTOC (Hirokawa et al., 1990). Myosins bind to actin forming actomyosin which has contractile properties (Bárány, 1967).

Membrane bending proteins: BAR-domain containing proteins (e.g., Bin/Amphiphysin/Rvs167 (Lee et al., 2002)) form dimeric banana-shaped alpha-helix coiled-coils and bind to cellular membranes facilitating membranes deformations. BAR domains can be classified into several subgroups: classical BAR/N-BAR, F-BAR, and I-BAR. N-BAR domain containing proteins are characterized by an additional N-terminal helix which promotes membrane curvature (Gallop et al., 2006). F-BAR domains are flatter than classical BAR domains allowing them to bind to larger liposomes (Frost et al., 2008). I-BAR (for Inverse BAR) domains facilitate negative membrane curvature (Mattila et al., 2007).

Membrane-constricting proteins: To pinch off an invagination from the rest of the membrane dynamin facilitates the constriction at the tubular neck of the invagination (Shpetner and Vallee, 1989). Dynamin is a GTPase that binds to membranes through a PH domain (Downing et al., 1994). Membrane fission is catalyzed through GTP hydrolysis (Mears et al., 2007).

Fusion machinery: Membrane fusion relies on SNAREs that are evolutionary-conserved molecules sharing a SNARE domain (also called SNARE motif) of about 60 residues constituting an α-helix (Weimbs et al., 1997). SNAREs are functionally classified as t-SNAREs localized on the target membrane and v-SNAREs localized on the vesicle (MSöllner et al., 1993). The interaction of t-SNAREs and v-SNAREs creates a so-called trans-SNARE complex (or SNAREpin) that is made of 4 SNARE motifs in a parallel four-helical bundle structure. The full zipping of the SNARE complex catalyzes the membrane fusion. After fusion, the zippered SNARE complex (so-called cis-SNARE) complex is disassembled by the N-ethylmaleimide-sensitive factor (NSF) and ***α***-soluble NSF-attachment protein (***α***-SNAP) through ATP hydrolysis (Ma et al., 2016). The recycling of v-SNAREs by vesicle budding closes the cycle (Rizo and Südhof, 2002).

Tethering/adapter molecules: The recruitment of *molecular motors* to membranes is performed through dedicated adaptors. Note that many adapter molecules interact with motors of both kinesin and dynein families (e.g., JIP4 (Willett et al., 2017), RILP, BicD2, FIPs). Accumulated evidence indicates that this step is regulated by small GTPases of the Rab family (see below). For instance, Rab11& Rab4 on recycling endosomes recruit FIP (Shiba et al., 2006) and WIP (Gryaznova et al., 2018), respectively; Rab5 on early endosomes recruits Rabaptin-5 (Stenmark et al., 1995); Rab27 on MVB/melanosomes recruits melanophilin that recruits the actin-dependent motor Myo5a (Strom et al., 2002); Rab7 on lysosomes recruits RILP (Cantalupo et al., 2001); Rab6 on the Golgi complex recruits BicD2 as well as the molecular motors KIF20A (rabkinesin) and MYH9 (myosin IIA), and Rab3 recruits synaptotagmin-like protein 4a (Slp4-a) that recruits MYH9 (Encarnação et al., 2016).

Small GTPases: Intracellular trafficking is regulated by several families of small GTPases, including the RAB, ARF, RHO, RAC family. These enzymes are anchored to membranes via a post-translational lipidation. Guanosine nucleotide Dissociation Inhibitors (GDIs) cover the lipid species of the GTPase in its GDP-, non-membrane-bound form. In their active, GTP-bound form, they recruit different effectors to membranes. They are activated by Guanine Nucleotide Exchange Factors (GEFs) that exchange a GDP to a GTP (Cherfils and Chardin, 1999), and deactivated by GTPase-Activating Protein (GAPs) that facilitate GTP hydrolysis to GDP (Das et al., 2001).

Box 2 **| Quantifying the organelle landscape**

Point pattern representing organelles in the cell, trees in the forest or stars in the galaxy, arise regularly in science. One of the fundamental questions about these point patterns is to infer the rules shaping their spatial structures and especially if they are uniformly randomly distributed, i.e., CSR. Spatial statistics tackle this challenge providing plethora of tools and among them the Ripley K function, is undoubtedly the queen (Dixon, 2014). It quantifies the average number of neighbors in a radius r around points. However, the higher the density of points, the more neighbors is expected. Hence, the number of neighbors is normalized by the density of points. In case of CSR, the Ripley K function is equal to $\pi r^{2}$ . Hence, values $>\pi r^{2}$ indicate more neighbors than expected, i.e., clustering and conversely values $\langle \pi r^{2}<$ indicate repulsion between points. Ripley K function has the great advantage to quantify spatial organization at different scales r and not to summarize the organization to a single number. For example, Ripley K function can detect clustering at a scale and see repulsion between clusters at a larger scale. As an illustration, we recently used it to demonstrate that lysosomal exocytosis is clustered at the whole cell scale (Lachuer et al., 2023) as others did for Golgi-derived exocytosis (Yuan et al., 2015; Urbina et al., 2018; Sebastian et al., 2006). Moreover, Ripley K function has many extensions to address more precise questions, e.g., a spatiotemporal version (Diggle et al., 1995) that we used to demonstrate that lysosomal exocytosis events clustered in time are more likely to be also clustered in space (Lachuer et al., 2023), or a multivariate version (Dixon, 2014) that have been used to demonstrate the co-clustering of H-Ras and K-Ras isoforms at the plasma membrane (Prior et al., 2003). These tools are available in different softwares including the Spatstat R package, which serves as the reference software (Baddeley et al., 2016). It appears that of the fundamental property of the organelle landscape is its non-random organization. In the past, organelle landscape has been traditionally described qualitatively but advances in imaging and in the popularization of these tools now allow a much more quantitative and rich description of the spatial rules governing the organelle landscape (Schauer et al., 2010; Ba et al., 2018).
